# Pathologic and hemodynamic changes of common carotid artery in obstructive sleep apnea hypopnea syndrome in a porcine model

**DOI:** 10.3906/sag-1807-170

**Published:** 2019-06-18

**Authors:** Yongyi LIU, Lu GAO, Weinong LV, Lin LIN, Yi WANG, Fan JIANG, Fan FENG

**Affiliations:** 1 The Fourth Affiliated Hospital of Jiangsu University, Jiangsu University, Zhenjiang China; 2 Medical College of Jiangsu University, Jiangsu University, Zhenjiang China; 3 The Huishan District Second People’s Hospital of Wuxi City, Wuxi China

**Keywords:** Sleep apnea obstructive, common carotid artery, hemodynamics, pathology

## Abstract

**Background/aim:**

To prepare a porcine model of obstructive sleep apnea-hypopnea syndrome (OSAHS) and observe the pathological and hemodynamic changes in the common carotid artery.

**Materials and methods:**

Twelve male miniature pigs were randomly divided into the model and control group (n = 6). Pigs in the model group were kept in an air-flow negative pressure chamber at 0.96 ± 0.01 kPa, and the air oxygen content, temperature, and humidity were kept at normal culture conditions in both groups. After pigs in the model group presented symptoms of OSAHS, changes in the hemodynamics and morphology of the carotid artery were analyzed using color Doppler, and light and electron microscopy.

**Results:**

An animal model of OSAHS was successfully created. The internal diameter of the carotid artery of pigs in the model group was decreased, while the intima thickness, peak-systolic mean velocity, and resistance index were increased when compared to the control group (P < 0.05). The results of the light and electron microscopy revealed an incomplete elastic plate, increased media thickness, irregular morphology of the smooth muscle cells, increased collagen fiber bundles, partially disordered elastic fibers, and smooth muscle layers. The quantitative analysis showed significantly increased elastic fibers in the media of the carotid artery in the model group (P < 0.01).

**Conclusion:**

Pathological changes in the tissue structure and hemodynamics in the negative pressure-induced pig OSAHS model were observed. We suggest that alterations in the upper airway pressure during OSAHS may lead to cardiovascular conditions through its pathological effects on the carotid artery.

## 1. Introduction

Obstructive sleep apnea-hypopnea syndrome (OSAHS) is characterized by repetitive episodes of airway reduction (hypopnea) or cessation (apnea) due to pharyngeal narrowing, leading to fragmented sleep cycles [u293a]. It was found that those who were male, of advanced age, and had higher body mass index had a higher prevalence of OSAHS [2]. Studies have looked at the aerodynamic properties of the pharynx in OSAHS, and a greater negative pressure in the pharyngeal cavity was shown to contribute to increased collapsibility of the pharynx [3]. Additionally, the pharynx has smaller average area and more compliance in OSAHS, leading to a narrower and more collapsible upper airway in OSAHS patients [4]. 

It has also been found that an increase of 1 unit in sleep apnea-hyponea index increased the risk of stroke by 6% in men [5]. Independent studies have shown that patients with OSAHS are at increased risk of ischemic heart disease, atherosclerotic coronary disease, diabetes, hypertension, congestive heart failure, acute coronary syndrome, stroke, and cardiovascular mortality [u293d]. Moreover, OSAHS patients have increased frequency of stroke and arterial fibrillation [10]. The application of continuous positive airway pressure to increase air pressure in the throat and prevent its collapse could reverse or attenuate OSA and its associated cardiovascular effects [u293e]. However, the mechanism of how OSAHS affects the cardiovascular system is still unclear.

Previous studies used rat models to investigate the effect of OSAHS on the cerebral cortex, skeletal muscle, and visceral fat tissues [u293f]. Studies have also employed a porcine model system to study the effect of OSAHS on the progression of chronic kidney disease [u2940]. As the size and cardiac physiology of porcines are similar to those in humans, this model system confers a huge advantage over other systems [u2941]. 

In this study, we attempted to probe the link between cardiovascular system and OSAHS by measuring changes in the structure and hemodynamic properties of the carotid artery during OSAHS in a porcine model. We measured several parameters, including the thickness of the intima, inner diameter of the carotid artery, peak-systolic mean velocity (S), and resistance index (RI) [15]. We investigated if the differences in the structure and hemodynamic properties of the carotid artery in OSAHS and normal cases could contribute to the development of cardiovascular conditions.

## 2. Materials and methods

### 2.1. Source and grouping of the experimental animals

A total of 12 male miniature pigs, aged 6 months and weighing 17 ± 1.5 kg, from Guizhou Province, Southwest China, were used. After habituation, the 12 pigs were randomly and equally divided into the model and normal control groups. All of the procedures and animal experiments were approved by the Animal Care and Use Committee of Jiangsu University.

### 2.2. Preparation of the low pressure chamber

A negative pressure chamber (as shown in Figure S1), measuring 1.5 × 2 × 3 m, was made of metal sandwich foam material, and the atmospheric pressure was kept at 0.96 ± 0.01 kPa. The air oxygen content was maintained at 22.5 ± 0.3%, and the temperature and humidity were maintained at 22–24 °C and 40%–50%.

### 2.3. Preparation of the OSAHS pig model

After 3 months of adaptive feeding, pigs in the model group were placed and fed in the negative pressure chamber for 6 months (time was chosen according to the results of our preliminary experiment, the pigs were kept in the chamber for 12-h day time including the time of feeding) to observe the activities, sleep, and snore decibel of the animals. The respiratory rate, respiratory waveform, and respiratory pressure were detected using the mechanical characteristics of the airway to assess if the pigs in the model group had symptoms similar to those of an OSAHS patient. The normal control group was fed in a similar chamber with the same conditions, except for a low negative pressure.

### 2.4. Hemodynamic measurements of the common carotid artery in the OSAHS model

Pigs in the model and control groups were anesthetized by an intramuscular injection of ketamine (2 mg/kg; The Pharmacy Department of the Fourth Affiliated Hospital of Jiangsu University, Zhenjiang, China). The porcine carotid artery diameter, intima-media thickness, S, diastolic end blood velocity (D), RI, carotid artery peak systolic velocity, and diastolic end blood flow velocity ratio (S/D) were measured using a United States Acuson-XP/10 EEG Doppler diagnostic instrument (Siemens, Beijing, China). The measurement site was located at the bifurcation of the common carotid artery.

### 2.5. Histological observation of the common carotid artery in the OSAHS model using light microscope

The miniature pigs were euthanized via carotid artery bleeding. The left carotid artery of each experimental pig was taken, 10 g of the fresh sample was fixed with 10% neutral formaldehyde solution (Sinopharm Chemical Reagent Co. Ltd., Shanghai, China), and the paraffin sections were prepared. The left carotid artery structure was observed using a PUS-BX51 OLYM optical microscope (Olympus Corporation, Shanghai, China). Using an HPIAS-1000 high definition color pathology image analysis system, the film thickness of the common carotid artery and the elastic fibers were quantitatively analyzed.

### 2.6. Electron microscopy

Another fresh sample of 1 cm3 was fixed with 2.5% cold glutaraldehyde (Sinopharm) and then 1% osmium tetroxide (Sinopharm) for 2 and 1 h, respectively. The specimens were dehydrated with graded acetone (30%, 50%, and 70%; Sinopharm), embedded with resin Epon-812 (Sigma, Shanghai, China), and located by semithin sectioning for 70-nm ultrathin sections. The sections were observed under a JEM-1200 transmission electron microscope after uranyl acetate (Sinopharm) and lead citrate double electron staining.

### 2.7. Statistical analysis

Data were processed and analyzed using SPSS 10 (IBM Corp., Armonk, NY, USA) statistical software. The data were represented as the mean ± standard deviation. T test was used to compare the model and control groups. P < 0.05 indicated statistical significance.

## 3. Results

### 3.1. Preparation of the OSAHS pig model

**Figure 1:**
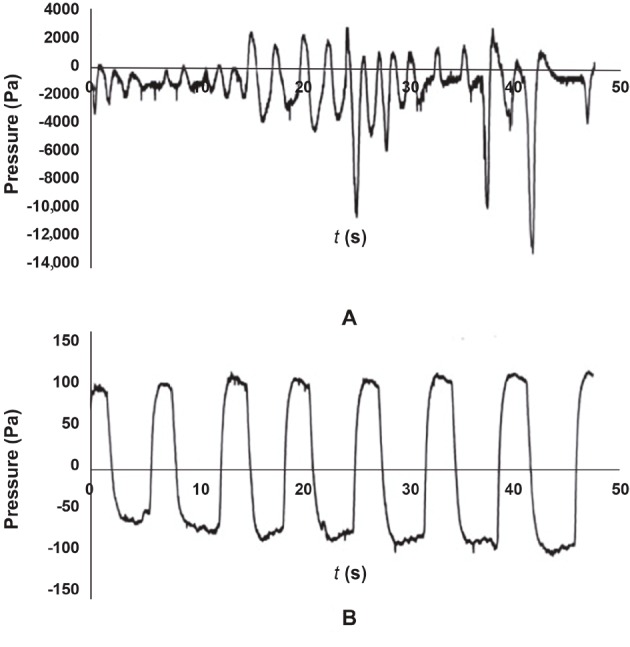
Respiratory pressure waveform of the model (A) and control (B) groups.

After eating and staying in the hypobaric chamber for 6 months, there was no significant difference in body weight with the same amount of diet between the control and the model groups (25.21 ± 1.12 kg vs. 24.89 ± 1.47 kg, P > 0.05). However, the miniature pigs in the model group showed reduced activity, increased sleeping time, snoring (43–51 dB), accelerated respiratory rate, negative respiratory pressure value (3.5 to 4 kPa), and an irregular pattern of respiratory pressure waveform, similar to OSAHS symptoms in humans (Figure 1A). The respiratory pressure waveform in the control group was normal (Figure 1B), indicating that the experimental group had OSAHS and could be used as a model system to investigate it.

### 3.2. Hemodynamic properties of the common carotid artery in the OSAHS model

The intima of the common carotid artery visualized using 2-dimensional ultrasound was smooth in the OSAHS pigs. The medial layer had low echo and reflectivity. The internal diameter of the common carotid artery was smaller, and its intimal thickening was significantly higher than in the control group (P < 0.05). The hemodynamic parameters of the carotid artery in the model group were measured by Doppler imaging. All 3 parameters (S, RI, S/D) were increased in the pigs with OSAHS, indicating increased vascular resistance when compared to the control group (P < 0.05) (Table 1)

**Table 1:** Ultrasonometry parameters of the common carotid artery in miniature pigs of the control and model group (n = 6).

		Control	Model	P-value
General morphology	Puncture resistance	Low	High		Thickness	561.53 ± 44.98 µm	655.16±35.59 µm	0.000
	Light microscope	Internal elastic lamina	Integrate	Not integrate		Intima media thickness	410.16 ± 23.76 µm	525.34 ± 49.82 µm	0.000	Elastic fiber in intima media	Regular arrangement	Elastic fiber hyperplasia with derangement	
Electron microscope		Morphology of smooth muscle cells	Regular	Irregular		Myofilament	Rich	Reduced		Macula densa	Clear	Reduced		Fibrinoid substances	Not seen	Focal distribution		Nuclear morphology	Regular	Irregular	

HR: heart rate, IMT: intimal-medial thickness, S: peak-systolic mean velocity, D: end-diastolic mean velocity, RI: resistance index. *P < 0.05, **P < 0.01 vs. the control group.

### 3.3. Light microscopy observations of the common carotid artery wall in the OSAHS model

**Figure 2:**
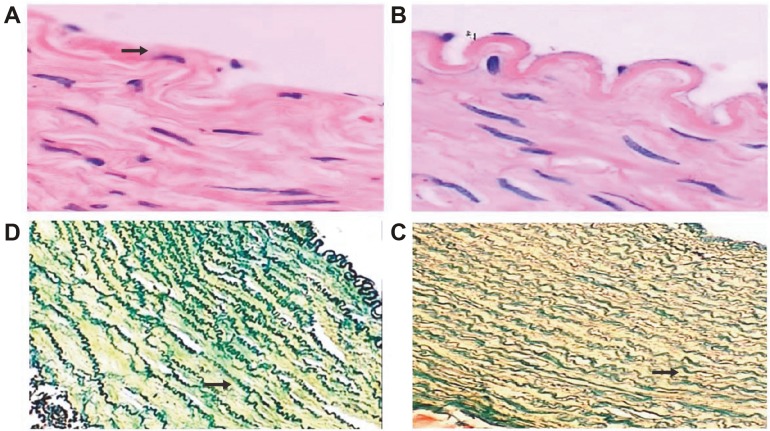
Light microscopy image of the endothelial tissues and elastic fibers in the left carotid artery of animals in the control and model group. A: Control group: endothelial cells were unclear and the inner elastic plate was incomplete (H&E, ×400); B: Model group: increased media thickness and hyperplasia of elastic fibers (V. G, ×200); C: Model group: clear endothelial cells with complete inner elastic plate (H&E, ×400); D: Control group: elastic fibers showed regular alignment in the tunica media of the left carotid artery (V. G, ×200).

In the model group, the common carotid artery wall was thicker, had poorer elasticity, greater toughness, and greater puncture resistance. In the control group, the artery tube wall was uniform, had good elasticity, punctured smoothly without resistance, and had no obvious lesions. Pigs in the model group had an artery tube wall thickness of 655.16 ± 35.59 µm. The inner layer of the carotid artery had irregular and sparse elastic fibers, and an incomplete internal elastic lamina (Figure 2B). The film thickness was 525.34 ± 49.82 µm, layered yet clear, had smooth muscle cells, and the nuclear morphology was irregular. The hematoxylin and eosin (H&E) staining showed the proliferation and disordered arrangement of the elastic fibers (Figure 2C). In the control group, the arterial wall thickness was 561.53 ± 44.98 µm and the carotid artery wall had a 3-layer structure, with the inner layer consisting of monolayer of endothelial cells, connective tissue, and internal elastic lamina. The endothelial cell morphology was normal and the elastic plate had a continuous wave shape (Figure 2A). The film thickness was 410.16 ± 23.76 µm, medial smooth muscle, and the elastic fibers had an ordered arrangement (Figure 2D); the external elastic plate was not obvious. The quantitative analysis of the elastic fibers (Table 2) showed that the total number of the elastic fibers, their surface number density, total area, and area percentage in the left carotid artery in the model group were significantly higher than those in the control group (P < 0.01).

**Table 2:** Quantitative analysis of elastic fibers in the left carotid canal of miniature pigs.

	Control	Model	P-value
Sum	409 ± 56	170 ± 35	<0.001
Pixel density	0.0057 ± 0.0079	0.0239 ± 0.0005	<0.001
Total area (A/µm2)	7175.9 ± 1660.8	13781.3 ± 473.2	0.004
Area percentage (%)	10.07 ± 2.33	19.34 ± 6.65	0.004
Total perimeter (l/µm)	8398.8 ± 1643.5	8397.8 ± 1447.4	0.099
Ratio of perimeter to area	1.48 ± 0.29	1.48 ± 0.25	0.975
Average space distance (l/µm)	25.02 ± 5.60	22.45 ± 5.67	0.407

### 3.4. Electron microscopy observations of the carotid artery wall in the OSAHS model

**Figure 3:**
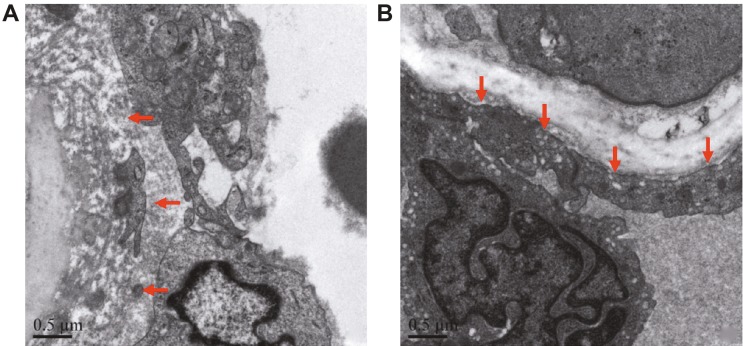
Electron microscope image of the left carotid artery of pigs in the model (A) and control (B) groups (×20,000).

Electron microscopy showed that the carotid endarterium had no obvious change, the collagen fiber bundles were increased, some elastic fibers and smooth muscles of the layered structure were disordered, the fibrinoid materials were focally distributed, and the smooth muscle cells had irregular morphology (Figure 3A). Moreover, the cell filaments were reduced, the nucleus was irregular, and red blood cell extravasation was visible near the endometrium. Electron microscopy of the control group showed that the morphology of the smooth muscle cell surface was rich with irregular cellular protrusions. There was abundant cytoplasm, a high content of myofilaments, clear macula densa, increased interstitial collagen fibers, and no observed inflammatory cell infiltration (Figure 3B). In the model group, hyperplasia, degeneration, and focal fibrinoid necrosis were found in the medial elastic fiber of the left carotid artery (Figure S2A). Degeneration and disordered arrangement was found in the medial smooth muscle of the left carotid artery (Figure S2B). For a comparison of the general morphology, the light and electron microscope observations of the left carotid artery wall in the control and model groups are shown in Table S1.

## 4. Discussion

In this study, we investigated the link between OSAHS and cardiovascular conditions associated with it. To that end, we assessed the differences in the structure and hemodynamic properties of the carotid artery in miniature pigs with OSAHS and control pigs. 

It was found that the internal diameter of the common carotid artery in the upper airway of the mini pigs with OSAHS was smaller, its elasticity was reduced, the intimal thickening was increased, and the elastic fibers were disordered. It was shown that the morphology of the smooth muscle cells in the left carotid artery was irregular with a focal deposition of the fibrinoid material in the model group. It was also shown that the hemodynamic parameters, including the D, RI, and ratio of systolic and diastolic pressure were increased in the model group. Thus, OSA and hypopnea caused by the upper airway obstruction lead to changes in the structure and hemodynamic behavior of the carotid artery during OSAHS. Previous studies have shown that assessment of the carotid artery is an important index for estimating the risk associated with coronary artery diseases [u2942]. Thus, we suggest that these changes in the carotid artery could explain the emergence of cardiovascular diseases.

Clinical studies have found endothelial dysfunction and arterial wall hardening in OSAHS [u2943], which are different from the physiological degeneration or other conditions caused by arterial diseases [u2944]. However, there is still a lack of relevant research regarding the effects of OSAHS on the cardiovascular system of patients. In this study, we successfully prepared an OSAHS mini pig model with the application of a negative pressure chamber, which has been previously shown to cause OSAHS [u2945]. We used the miniature pig model system because the coronary circulation and arterial anatomy of pigs and humans are very similar. Moreover, pigs offer the best model to study plaque instability, as they have a similar lipoprotein profile to that of humans [u29460]. 

Abnormal pharyngeal airway pressure is one of the critical factors in the pathogenesis of OSAHS. When the upper airway is in a negative pressure state, the soft tissue of the pharyngeal cavity gradually undergoes hyperplasia, hypertrophy, and reconstruction, causing the pharyngeal cavity to narrow and reduce the effective respiratory cross section area. This causes a further lack of oxygen, promoting OSAHS formation and development [7]. Studies have also enabled a reversal of the symptoms of OSAHS by either changing the sleeping position or by the application of a continuous positive airway treatment [u29471]. Previous studies housed SD rats in sealed cabins in a hypoxic environment, leading to reconstruction of the soft palate and snoring [u2948,u29490]. It was found that the structure and function of the pharynx in rats was similar to the pathological changes of OSAHS after the induction of OSAHS by low pressure hypoxia [u294a]. In this study, we constructed an OSAHS model of Guizhou miniature pigs using a controlled low pressure module and observed the hemodynamic and histopathological characteristics of the common carotid artery. We also showed that the symptoms of the pig model in the low pressure chamber environment were similar to those of OSAHS; thus, miniature pigs can be employed as an effective OSAHS disease model.

However, we only observed the hemodynamics and pathological changes of the common carotid artery, and OSAHS is a systemic disease, where long-term hypoxia only causes damage to the cardiovascular system, triggering atherosclerosis, sudden death, cardiovascular and cerebrovascular accidents, hypertension, and other diseases, but it can also lead to neurological damage, such as nerve cell stress response, apoptosis, and degeneration. Furthermore, our observations were limited to changes in the vascular morphology, and the potential molecular signaling pathways were not further discussed. Further studies are required to separate the vascular endothelial cells and smooth muscle cells in the experimental and control group to detect the differential expression of mRNA, miRNA, lncRNA, or protein, and verify the downstream molecular targets. Meanwhile, the evidence also showed that gut microbiota contributed to the hypertension observed in OSA [u294b], this indicated that gut microbiota may have been an influential factor in inducing OSAHS diseases. However, the study also showed that OSAHS could alter gut microbiota [u294c]; hence, future experiments could be designed to verify the direct causal relationship between gut microbiota with OSAHS.

In this study, the structure and hemodynamic characteristics of the carotid artery in mini pigs with OSAHS and a control were compared to investigate the link between cardiovascular diseases and OSAHS. It was shown that miniature pigs can be employed as a model system for OSAHS by keeping them in low pressure chamber, which lead to the reconstruction of the upper airway and thus, OSA and hypopnea. It was also observed that the common carotid artery in the upper airway of mini pigs with OSAHS had a thinner internal diameter, reduced elasticity, disordered elastic fibers, and increased intimal thickening. The hemodynamic parameters of the carotid artery (D, RI, ratio of systolic and diastolic pressure) were also increased. We speculated that these changes in the carotid artery properties could lead to increased cardiovascular conditions. Thus, this study provides a link between cardiovascular diseases and OSAHS; however, further studies are required to identify the downstream molecular targets of inducing negative pressure in the upper airway.

## Acknowledgments

This study was supported by the National Natural Science Foundation of China (11472118), the Maternal and Child Health Research Project of Jiangsu Province (F201604), and the Science and Technology Development Fund of Wuxi City (WX18IIAN044).
